# The Wedding-Day

**Published:** 1872-07

**Authors:** 


// # HALL'S
### JOURNAL OF HEALTH.

Vol. XIX.—JULY, 1872.—No. VII.

### THE WEDDING-DAY.

Two healthy persons, with trusting, loving hearts, having been united in marriage, immediate preparation should be made for housekeeping; following the beautiful instincts of the birds of the forest, whose greatest happiness seems to be in preparing a place in which they may nestle with their young. The very labor necessary may well be supposed to be one of love and delight.

For the young pair to enter a splendid mansion, completely and elegantly furnished by parental love, the very day after marriage, does not afford the thousandth part of the pure, enduring, and healthful gratifications which attend those, seemingly less favored by fortune, whose home has to be selected with much previous calculation, and debating, and hesitancy; where every article of furniture has to be talked over; its style and quality to be considered; what amount of means can be afforded to procure this, that, and the other housekeeping necessity? can the money be spared to obtain that elegant pattern of carpet? would it not be better to take something less costly for a year or two, then move that up-stairs, and have the more elegant one for the parlor? The very circumstance of having to stop for want of funds long before the furnishing is completed, when in a plain way, is not without its advantages, its springs of lovingness; for these things bring the young husband and wife to counseling together; the wife's native pride and fine taste, and the young man's prudence, balancing against each other. His devotion urges him to gratify the woman whose happiness is his highest aim, and his secret thought, that a little more energy, a little more application, a little longer staying at his place of business, will enable him to make it up: then comes the fear of debt; of being hampered; of possible failure of this, that, or the other plan. On the other hand, the wife fears that he cannot afford it; that it may require him to work too hard; then comes the indefinite apprehension of sickness and suffering, and all to gratify her; and she resolves to do without it. He insists that she ought to have it; and then they begin to skirmish, and make their little feints and pretenses, and practice their filmy infinitesimal falsities, to the end that the woman eventually has her way in the first battle of married life: she stoops to conquer; she governs in the future by yielding now, resolving that she can do without the coveted article for the present; telling him that in a short time it may be better and more safely afforded. The young man straightway looks upon the blossom before him with a greater devotion, a deeper, purer, warmer love, and resolves in his own mind that she is worthy of all the efforts he can make for her happiness, and that all the energies within him shall be exerted with a will to gratify every desire of her heart; and thus, before they know it, they have been wedded together in a closer, stronger bond than any clerical formula ever forged; for in this they have learned to "take counsel together," to defer to each other's views, and arguments, and wishes. Each has seen in the other a disposition to mutual sacrifices, and a habit of giving up one's own will for the gratification of the other is begun, and one of the broadest stones for the foundation of domestic happiness is laid ; and then, the one who has given up is more than repaid by the conqueror, who feels within a purer life and a deeper emotion arising, to go out in acts of lovingness which make both giver and receiver happier and better.

Working thus together, playing into each other's hands, striving to accomplish any commendable object which is to make both happier, which is to add to the common store making mutual sacrifices; bringing constantly into play each other's sympathies by labors, and efforts, and self-denials — these are things which bind young hearts together, and build up between them an affection, a love, a devotion, which passing years but purify and consolidate and sweeten until life's close.

This mode of beginning married life has other advantages. It gives opportunity for the exercise of hospitalities towards friends. This stimulates to greater industries, to tidiness in housekeeping, to neatness of attire, to the practice of little economies, to the exhibition of courtesies ; and to those little "praisings up" to guests, which will involuntarily escape from the lips of the new housekeepers, and which very things deepen attachments, fan anew the flame of love, and become another spring of domestic beatitudes.

Another high advantage is, housekeeping keeps the young wife's mind busy — a very important consideration. She is not only busy thinking, but it is a thinking on practical matters, on things necessary to be done promptly, yet with deliberation and judgment. Thus the powers of the mind are evolved, responsibility is exercised, executive ability is brought into requisition, self-reliance is cultivated, because the husband is not at hand to be consulted ; and when he comes home and finds she has acted with judgment, with prudence, with wisdom, he shows his appreciation of it by his cordial and affectionate commendations, and the light breaks in upon her for the first time that she is not a doll, a plaything, a baby, or a child, but that she has capabilities. Then she feels stronger ; there is a consciousness that she can be a help in the family; that she is worth something; that while she is a recipient, she can be an aid. The husband, seeing this practical exhibition of her capacities, is gradually led to ask her advice, to talk with her about his own business matters, for the sake of possible advantages to be derived from her suggestions. Thus one leans on the other; they look up to each other; mutual and additional confidences arise, and it is not long before they find that, between the household affairs of the wife, in which she sometimes wants her husband's counsel, and the more important business matters of the husband, about which he is quite willing to listen to her suggestions and hints, they have plenty to talk about. There is no sitting in the room for half an hour at a time without the exchange of a single word; there is no silent smoking of a cigar for a great part of the evening; no poring over a novel by the hour; no burying the nose in a newspaper, until every column has been read, advertisements and all; no sudden jumping up from a chair to take a little walk, to visit a friend, to meet a business appointment, — the wife left alone all the while to brood over the mishaps, the annoyances, the disappointments of the day. On the contrary, each has subjects of inquiry; each has points of information to communicate, and a mutual interest in each other's respective departments springs up, which still further tightens the marriage bond. Each hour and each day is filled with its own duties, and responsibilities, and satisfactions, and there is domestic happiness, more thoroughly cemented every day and every hour, and giving the promise of a deeper and a purer enjoyment in the future.

One of the great faults and dangers of the times, especially in cities, is the increasing custom of young married persons spending the first months or years of married life in boarding-houses and hotels, or in the family of one of the parents. Trouble may be saved by this, the trouble of housekeeping; money may be saved by it; but it is at the expense of domestic comfort, of domestic happiness; more, it is risking domestic discord and domestic ruin; discord, because the young wife has nothing to do but to eat, and dress, and sleep, and lounge about; lolling on sofas, gazing out of front windows; frittering away the time in trifling conversation with callers as idle as herself; spending many hours in dreamy imaginings; in poring over worthless novels; making questionable acquaintances; at other times indulging in the vain ambitions which want of occupation and undesirable companionship engender, to say nothing of the bickerings, the suspicions, the envies, the jealousies, the misunderstandings, and the thousand other sources of disquietude and discontent which are found under any roof which covers more than one family.

Remembering that the prevailing condition of the mother's mind during pregnancy will be impressed upon the child that is born, the highest appeal possible is made to parental justice, humanity, and love to cultivate those feelings and affections and sentiments and thoughts which most ennoble our nature, by avoiding idleness during gestation; by keeping the mind on the alert, as much as possible, in housekeeping and domestic duties; in callings which keep the wife out of doors at least two or three hours every day, so that she shall be engaged in pleasurable activities to the extent of having the mind fully occupied, and the body engaged in doing something profitable, useful, pleasurable, with absolutely not a moment's leisure for nurturing dissatisfactions, envyings, remorses, hurt feelings, supposed slights or animosities of any description. But this is not half the duty; there is a positive obligation to engage in whatever may cherish and cultivate all the higher feelings of our nature, — our magnanimity, our benevolence, our love. Contemplate nothing that is not agreeable, that does not wake up the better sentiments. Gaze by the hour upon paintings, upon sculpture, on the waterfall and the mountain-top, on landscapes of tree and fountain, of fields and flowers, of lake and river, of hill and valley, listening to the songs of birds, to the music of the sweetest instruments, and that nearer the Divine, the human voice itself.

In addition to these, give many thoughts to religious contemplations, to inward reflections, to yearnings for human ameliorations, the relief of the poor, and the elevation of all.

The remarks made, and those to be made, are intended to be carried out in practice, not merely for the first, but for all subsequent gestations, during which periods it is the husband's highest duty, and without the performance of which, for every hour of every successive day, he cannot be a man, to make his wife's happiness his constant study; and in no way can he do this better than by a prompt gratification of every reasonable desire. Anticipate her probable wants; do everything with a prompt and cheerful alacrity; be quick in sympathies; plan pleasant surprises; make coming home, when the business of the day is done, an event lovingly looked for; come home full of news; bring messages from friends; never come empty-handed; a bunch of grapes to-day, a rare fruit to-morrow, next day a pink, or rose, or little flower; anything to show, without telling it, that the wife at home has been lovingly thought of. But there is more to be done than all this. The pregnant wife, in every word and tone and look and gesture, should see that her husband's heart is full of tenderness, that he has the chivalry of a lover, and that his whole deportment towards her is manliness itself. By no possibility, whatever might be the provocation, should an impatient look, or cross word, or angry reply ever escape him; and let every one see that the wife is considered the queen of the table, the mistress of the mansion. In these ways she will be kept occupied fully; will be kept hopeful, and will enjoy everything. She will also become self-respectful, self-appreciating, self-asserting, fearing nothing, all the time full of implicit trustingness in her husband's confidence and sympathies, pouring out upon him, in turn, the full measure of a woman's love; thus will the mother mould the moral character of her first-born in a cast which is noble, generous, and beautiful.

